# HLA polymorphisms and detection of kaposi sarcoma-associated herpesvirus DNA in saliva and peripheral blood among children and their mothers in the uganda sickle cell anemia KSHV Study

**DOI:** 10.1186/1750-9378-5-21

**Published:** 2010-11-18

**Authors:** Mercy Guech-Ongey, Murielle Verboom, Ruth M Pfeiffer, Thomas F Schulz, Christopher M Ndugwa, Anchilla M Owor, Paul M Bakaki, Kishor Bhatia, Constança Figueiredo, Britta Eiz-Vesper, Rainer Blasczyk, Sam M Mbulaiteye

**Affiliations:** 1 Infections and Immunoepidemiology Branch, Division of Cancer Epidemiology and Genetics (DCEG), National Cancer Institute, Bethesda, Maryland, USA; 2 Institute for Transfusion Medicine, Hannover Medical School, Hannover, Germany; 3 Biostatistics Branch, Division of Cancer Epidemiology and Genetics (DCEG), National Cancer Institute, Bethesda, Maryland, USA; 4 Institute of Virology, Hannover Medical School, Hannover, Germany; 5 Makerere College of Health Sciences, Kampala, Uganda; 6 Case Western Reserve Univ., Cleveland, OH, USA

## Abstract

Kaposi sarcoma-associated herpesvirus (KSHV, also called Human herpesvirus 8 or HHV8) is a γ-2 herpesvirus that causes Kaposi sarcoma. KSHV seroprevalence rates vary geographically with variable rates recorded in different sub Sahara African countries, suggesting that effects of genetic and/or environmental factors may influence the risk of infection. One study conducted in South Africa, where KSHV seroprevalence is relatively low, found that carriage of human leukocyte antigen (HLA) alleles HLA-A*6801, HLA-A*30, HLA-A*4301, and HLA-DRB1*04 was associated with increased shedding of KSHV DNA in saliva. Confirmation of those results would strengthen the hypothesis that genetic factors may influence KSHV distribution by modulating KSHV shedding in saliva. To explore these associations in another setting, we used high resolution HLA-A, B, and DRB1 typing on residual samples from the Uganda Sickle Cell Anemia KSHV study, conducted in a high KSHV seroprevalence region, to investigate associations between HLA and KSHV shedding in saliva or peripheral blood among 233 children and their mothers. HLA-A and HLA-DRB1 alleles were not associated with KSHV shedding in our study, but our study was small and was not adequately powered to exclude small associations. In exploratory analyses, we found marginal association of KSHV DNA shedding in saliva but not in peripheral blood among children carrying HLA- B*4415 and marginal association of KSHV DNA shedding in peripheral blood but not in saliva among children carrying HLA- B*0801 alleles. The contribution of individual HLA polymorphisms to KSHV shedding is important but it may vary in different populations. Larger population-based studies are needed to estimate the magnitude and direction of association of HLA with KSHV shedding and viral control.

## Findings

Kaposi sarcoma-associated herpesvirus (KSHV, also called Human herpesvirus 8 or HHV8) is a γ-2 herpesvirus etiologically linked to Kaposi sarcoma (KS) [1]. KSHV seroprevalence in the general population is highest in sub-Saharan Africa (50-60%), intermediate in South America and the Mediterranean countries (10-40%), and low in Europe and North America (< 5%) [2], suggesting that genetic and/or environmental factors may influence KSHV seropositivity patterns [3,4]. Environmental factors, including plants in regions of Africa where KSHV prevalence and KS incidence are high, have been postulated to influence KSHV lytic replication and, thus, increase KSHV shedding [5]. Colluzi *et al*., speculated that KSHV transmission is increased indirectly when KSHV infected saliva is used to soothe itchy insect bites caused by blood-sucking arthropods [6]. The role of genetic factors is less well understood. An effect of a major recessive gene on KSHV susceptibility or resistance to KSHV infection was postulated, based on statistical analysis of familial clustering of KSHV infection, among Noire-Marrons families in French Guiana [7]. Recently, a study in South Africa found increased KSHV DNA shedding in saliva among subjects carrying human leukocyte antigens (HLA) HLA-A*6801, HLA-A*30, HLA-A*4301, and HLA-DRB1*04 alleles [8]. Given that HLA polymorphisms have been shown to influence transmission, control, and pathogenesis of other viral infections such as human papillomavirus, human immunodeficiency virus type 1 and human T cell lymphotropic virus type 1 [9-11], this finding is biologically plausible. Thus, we sought to replicate the findings from South Africa in Uganda and to explore novel HLA allele associations with KSHV shedding using residual samples and data from the Uganda Sickle Cell Anemia KSHV study [12].

The Uganda Sickle Cell Anemia KSHV study included 600 children with sickle cell anemia, but without KS, who were enrolled from November 2001 to April 2002 at the Sickle Cell Clinic at Mulago Hospital [12]. The mothers of the children were also included when available. Children were confirmed by gel electrophoresis to have sickle cell anemia (homozygous for the sickle cell gene) and the mothers were presumed to have the sickle cell trait (heterozygous for the sickle cell gene), but not sickle cell anemia. Children and their mothers were tested serologically for anti-KSHV antibodies using two peptide enzyme immunoassays (EIA) to the K8.1 and ORF73 KSHV peptides, as previously described [12]. A subset of 233 children, including 183 who were KSHV seropositive on either the K8.1 or the ORF73 EIA and 50 children randomly selected from among 417 children who were seronegative on both assays, were further studied for KSHV viral shedding in saliva and in peripheral blood using quantitative polymerase chain reaction (qPCR) for KSHV DNA [3].

We performed high-resolution HLA-A, -B and -DRB1 typing on residual DNA obtained from saliva or buffy coat of the children and their mothers from the KSHV viral study. Alleles were separated by a group-specific amplification approach using multiple amplification primer mixes in parallel (PROTRANS S3/S4 HLA SINGLE ALLELE SEQUENCING SET, PROTRANS MEDICAL DIAGNOSTICS, HOCKENHEIM, GERMANY) [13]. The presence or absence of a PCR product was demonstrated by an agarose gel-based read-out for HLA-A and HLA-B while the PCR product detection for HLA-DRB1 was achieved by fluorescence-based read-out using the 5' nuclease technology [14]. Purification of the PCR product was performed enzymatically by exonuclease/shrimp alkaline phosphatase treatment (ExoSAP-IT; USB; Cleveland, OH, USA), followed by forward and reverse sequencing of exon 2 and 3 for HLA class I and of exon 2 for HLA class II, using Big Dye Terminator Technology (Applied Biosystems, Foster City, CA, USA). The sequencing reaction products were purified using the Montage SEQ96 sequencing reaction cleanup kit (Millipore, Billerica, MA, USA) and subjected to electrophoresis on a 3730 Genetic Analyzer (Applied Biosystems). The data were analyzed using the Sequence Pilot program (version 3.0; Protrans). Sequencing-based typing allowed determination of HLA allele groups on basis of resolution at the 2- digit level and alleles on basis of resolution at the 4-digit level [8].

We calculated the weighted distribution of HLA-A, -B, and -DRB1 allele group frequencies among the children and their mothers separately. The prevalence of allele groups was weighted back to the original study population. Thus, the weighted prevalence of HLA allele group (X) in the children = (prevalence of allele group X in KSHV-negative children × proportion of children who were KSHV negative) + (prevalence of allele group X in KSHV-positive children X proportion of children who were KSHV positive). The prevalence of HLA allele groups for the mothers was weighted back to the original study population based on KSHV status of the children because the mothers were selected when their child was selected.

We assessed associations between HLA allele groups and KSHV DNA detection for the alleles selected *a priori *based on previous associations with KSHV shedding in saliva: HLA-A*6801, HLA-A*30 and HLA-A*4301, and HLA-DRB1*04 separately for the children and their mothers. We also performed exploratory analyses to identify new associations. Odds ratios (ORs) for association and corresponding 95% confidence intervals (95% CIs) were computed using logistic regression models. We adjusted for KSHV serostatus by including the posterior probability of KSHV infection estimated using multivariate mixture models as previously described [15]. All statistical tests were two-sided and *p*-values < 0.05 were considered statistically significant.

Among 233 children with DNA, HLA was successfully typed for the A locus in 223, for the B locus in 225, for the DRB1 locus in 223 children. Among 233 mothers with DNA, HLA was successfully typed for the A locus in 226, for the B locus in 226, for the DRB1 locus in 227 mothers. The weighted prevalence for HLA loci, HLA-A, -B, and -DRB1 and the number of people carrying the allele group for children and their mothers separately are shown in Table 1. The allele group distribution between mothers and children for HLA-A, -B, and -DRB1 were similar (Table 1). For each locus, the 4 most frequent allele groups were HLA-A*02, A*30, A*68, A*74; B*58, B*53, B*15, B*42; and DRB1*11, DRB1*15, DRB1*13, DRB1*01 among the children and HLA-A*02, A*30, A*68, A*23; B*58, B*45, B*15, B*42; and DRB1*11, DRB1*15, DRB1*13, DRB1*03 among the mothers. HLA-A*43, which was associated with KSHV shedding in South Africa, was not observed in our study. HLA-DRB1*04 allele group was observed in only 1% of the children and in only 2% of the mothers.

**Table 1:** Weighted* HLA allele group prevalence among children and mothers in the Uganda Sickle Cell Anemia KSHV Study, 2001-2002

	Children		Women		
**HLA group**	**Number typed†**	**Percent positive for allele group (number positive)**	**Number typed†**	**Percent positive for allele group (number positive)**	***p-value *‡**

HLA-A	223		226		
*01		5.1 (22)		3.5 (22)	
*02		19.5 (82)		20.0 (78)	
*03		5.2 (23)		4.6 (28)	
*23		10.2 (42)		8.8 (38)	
*29		5.0 (32)		7.3 (37)	
*30		12.0 (75)		13.7 (82)	
*36		5.4 (13)		5.2 (13)	
*66		5.0 (26)		8.0 (36)	
*68		11.5 (39)		10.6 (43)	
*74		10.7 (38)		6.2 (31)	
					0.94
HLA-B	225		225		
*07		4.9 (18)		6.2 (18)	
*08		3.4 (18)		3.1 (15)	
*15		10.5 (59)		9.0 (65)	
*42		6.8 (35)		8.9 (40)	
*44		3.9 (14)		3.1 (16)	
*45		8.0 (32)		11.9 (41)	
*49		1.5 (13)		5.3 (16)	
*53		11.6 (40)		8.7 (36)	
*57		5.9 (21)		2.1 (22)	
*58		16.6 (59)		16.8 (73)	
*82		6.1 (6)		0.1 (2)	
					0.98
HLA-DRB1	223		227		
*01		10.5 (48)		8.0 (37)	
*03		9.4 (56)		10.1 (59)	
*04		1.0 (4)		1.7 (4)	
*07		6.5 (31)		5.8 (37)	
*08		5.0 (20)		5.6 (20)	
*11		28.2 (113)		28.8 (116)	
*12		3.7 (11)		6.1 (17)	
*13		11.7 (68)		12.3 (59)	
*15		15.9 (61)		14.4 (70)	
					0.95

* For both children and their mothers prevalence for HLA-A, -B, -DRB1 allele groups was weighted according to KSHV serostatus of the children (the selection criteria for inclusion in the study) using the formula: weighted prevalence (α) of HLA allele group X in the children (or mothers) = (Prevalence of X in KSHV negative children (or mothers) × proportion of KSHV negative children) + (Prevalence of X in KSHV positive children (or mothers) × Prevalence of KSHV positive children). See methods in text for details.

† Total is less than 233 because fewer subjects were successfully typed for HLA-A, -B and -DRB1

‡ P-value for heterogeneity comparing HLA-A, -B and -DRB1 allele group distribution between mothers and children

HLA-A*3001, HLA-A*3002, HLA-A*6801, HLA-A*6802, and DRB1*0405 alleles were not associated with detection of KSHV DNA in saliva or in peripheral blood in the children (Table 2) or in the mothers (results not shown). We found increased risk of detectable KSHV DNA in saliva among children carrying at least 1 HLA- B*4415 allele (OR 5.5; 95% CI 1.1-28.6), but the risk was not increased for detecting KSHV DNA in peripheral blood (OR 1.1; 95% CI 0.1-9.2). We found increased risk of detectable KSHV DNA in peripheral blood in children carrying at least 1 HLA- B*0801 allele (OR 3.9; 95% CI 1.4-10.8), but the risk was not increased for detecting KSHV DNA in saliva (OR 1.1; 95% CI 0.3-4.1).

**Table 2:** Association between HLA alleles and KSHV DNA detection in saliva and peripheral blood among children in the Uganda Sickle Cell Anemia KSHV Study, 2001-2002

Allele	# KSHV DNA positive/#KSHV DNA negative	OR (95% CI)	*P*-value	# KSHV DNA positive/#KSHV DNA negative	OR (95% CI)	*P*-value
Saliva *A priori*-motivated analyses				Peripheral blood		
HLA-A*30						
Absent	20/121	1		26/130	1	
At least 1 *3001	5/29	1.1 (0.5-4.3)	0.5	3/23	0.6 (0.2-2.2)	0.8
At least *3002	9/24	1.8 (0.8-4.1)	0.2	7/34	1.1 (0.5-2.7)	0.9
						
HLA-A*68						
Absent	30/139	1		31/153	1	
At least1*6801	0/6	0.0	0.6	1/5	1.0 (0.1-9.2)	1.0
At least1*6802	4/29	0.5 (0.1-1.8)	0.3	4/29	0.7 (0.2-2.3)	0.6
						
HLA-DRB1*04						
Absent	32/173	1		34/187	1	
At least 1*0405	1/3	1.8 (0.1-5.2)	0.5	2/2	5.5 (0.7-40.3)	0.1

Explorative analyses						
HLA-B*08						
Absent	31/160	1		29/176	1	
At least 1*0801	3/14	1.1 (0.3-4.1)	0.9	7/11	**3.9 (1.4-10.8)**	**0.01**
						
HLA-B*44						
Absent	30/165	1		33/176	1	
At least 1*4403	1/6	1.1 (0.1- 9.9)	0.9	2/6	1.9 (0.3-9.1)	0.2
At least 1*4415	3/3	**5.5 (1.1-28.6)**	**0.02**	1/5	1.1 (0.1-9.2)	0.9

KSHV: Kaposi-sarcoma-associated herpesvirus; OR: odds ratio; 95% CI: 95% confidence interval; HLA-A, -B, -DRB1: Human leucocyte antigen -A, -B, -DRB1

Our study did not replicate HLA associations with KSHV DNA shedding reported in South Africa [8]. Our null results are likely due to the small size of our study and hence limited power to demonstrate weak associations. Another reason is that, our study included a highly selected group of children, i.e., with sickle cell anemia and their mothers with sickle cell trait, who may be systematically different in their HLA genotype distribution from the general population. In addition, because KSHV shedding is often intermittent, associations between KSHV shedding and HLA may have been missed by our study that relied on peripheral blood and saliva, taken at one time point. We found that the distributions of HLA allele groups in the mothers and children were similar and resembled the distributions of HLA allele groups in two general population-based studies in Uganda [16,17] suggesting that the selected nature of our population may not be a major reason for our null results. Possibly, different alleles influence KSHV shedding in different populations, as suggested by the rare-allele advantage model [18]. The polymorphic nature of HLA is critical for conferring diverse and effective pathogen response. Because polymorphisms in HLA are influenced by pathogens that are common in the environment where the population resides and is well adapted, differences in haplotype structure in populations residing in geographically dispersed regions, such as Uganda and South Africa, could lead to variable HLA pathogen associations [19]. For example, HLA allele polymorphisms HLA- B*5301, HLA- DRB1*1302, and HLA- DRB1*0101 have been associated with genetic resistance [20-22] or HLA-DRB1*04 with susceptibility [23] to malaria, but the distribution is different in different populations where malaria is endemic [19,20,24]. In the study by, Alkharsah *et al*., [8], risk for KSHV shedding in saliva was increased among carriers of HLA-A*43, but this allele group was observed in 2.8% of women in their study. This allele group was not observed in our study and it was absent in another study that evaluated HLA polymorphisms in Uganda [17] and, thus, its effect cannot be replicated in Uganda. Taken together, ours and Alkharsah's study [8] provide the first insights about HLA associations with KSHV DNA shedding but additional studies are warranted to clarify the role of host genetics in KSHV epidemiology.

The strengths of our study include assessment of HLA polymorphisms on three loci among children and their mothers. The pre-selected sample of children with sickle cell disease, one time sampling of subjects and small sample size were limitations. Thus, it is possible that play of chance and multiple testing in small studies could explain findings from both ours and Alkharsah's studies. Large and better designed studies are warranted to evaluate the contribution of individual HLA polymorphisms to KSHV shedding in different populations.

## Competing interests

The authors declare that they have no competing interests.

## Authors' contributions

MGO analyzed and interpreted data, and drafted the manuscript. MV, TFS, BEV, CF, and RB used high resolution technique for HLA-A, B, and DRB1 allele typing. RMP contributed to analysis and interpreted data. CMN, AMO, PMB, interpreted data and edited the paper, KB interpreted data. SMM conceived the idea, guided data analysis, interpreted data and edited the paper. All authors had access to data, commented on and contributed to the final draft of the manuscript. All authors read and approved the final paper.
